# A new instrument to measure high value, cost-conscious care attitudes among healthcare stakeholders: development of the MHAQ

**DOI:** 10.1186/s12913-020-4979-z

**Published:** 2020-03-02

**Authors:** Serge B. R. Mordang, Karen D. Könings, Andrea N. Leep Hunderfund, Aggie T. G. Paulus, Frank W. J. M. Smeenk, Laurents P. S. Stassen

**Affiliations:** 1 Department of Educational Development and Research, School of Health Professions Education, Maastricht University, P. O. Box 616, 6200 MD, Universiteitssingel 60, 6229 ER Maastricht, the Netherlands; 2 Department of Neurology, Mayo Clinic, Rochester, MN USA; 3 Department of Health Services Research, Care and Public Health Research Institute, Maastricht University, Maastricht, the Netherlands; 4 Department of Pulmonary Medicine, Catharina Hospital, Eindhoven, the Netherlands; 5 Department of Surgery, Maastricht University Medical Center, Maastricht, the Netherlands

**Keywords:** High-value cost-conscious care, Attitudes, Instrument development, Learning environment, Post-graduate medical training

## Abstract

**Background:**

Residents have to learn to provide high value, cost-conscious care (HVCCC) to counter the trend of excessive healthcare costs. Their learning is impacted by individuals from different stakeholder groups within the workplace environment. These individuals’ attitudes toward HVCCC may influence how and what residents learn. This study was carried out to develop an instrument to reliably measure HVCCC attitudes among residents, staff physicians, administrators, and patients. The instrument can be used to assess the residency-training environment.

**Method:**

The Maastricht HVCCC Attitude Questionnaire (MHAQ) was developed in four phases. First, we conducted exploratory factor analyses using original data from a previously published survey. Next, we added nine items to strengthen subscales and tested the new questionnaire among the four stakeholder groups. We used exploratory factor analysis and Cronbach’s alphas to define subscales, after which the final version of the MHAQ was constructed. Finally, we used generalizability theory to determine the number of respondents (residents or staff physicians) needed to reliably measure a specialty attitude score.

**Results:**

Initial factor analysis identified three subscales. Thereafter, 301 residents, 297 staff physicians, 53 administrators and 792 patients completed the new questionnaire between June 2017 and July 2018. The best fitting subscale composition was a three-factor model. Subscales were defined as *high-value care*, *cost incorporation,* and *perceived drawbacks.* Cronbach’s alphas were between 0.61 and 0.82 for all stakeholders on all subscales. Sufficient reliability for assessing national specialty attitude (G-coefficient > 0.6) could be achieved from 14 respondents.

**Conclusions:**

The MHAQ reliably measures individual attitudes toward HVCCC in different stakeholders in health care contexts. It addresses key dimensions of HVCCC, providing content validity evidence. The MHAQ can be used to identify frontrunners of HVCCC, pinpoint aspects of residency training that need improvement, and benchmark and compare across specialties, hospitals and regions.

## Background

Providing high value, cost-conscious care (HVCCC) is critical to improve the value of health care and at the same time counter rising costs, eliminate wasted spending, and reduce overuse (provision of healthcare services with no medical basis or for which harms equal or exceed benefit) [1–5]. Value in this context can be understood as quality divided by cost over time [6]. Cost-conscious refers to the awareness an individual has on the specific expenses and cost-effectiveness of an intervention, as well as negative consequences as a result of providing – or not providing - an intervention, like patient dissatisfaction [7, 8]. Providing HVCCC requires physicians to balance the potential benefits and harms of a test or treatment, while simultaneously considering costs and possible drawbacks [7]. Physician practice patterns influence the number and type of healthcare services patients receive [9]. The post-graduate training appears to be particularly formative in shaping residents’ current and future behaviors related to high-value care, such as during exposure to faculty discussions on patient care [10]. Medical education thus has an obligation to ensure that stakeholders within the post-graduate learning environment support the development of HVCCC practice patterns [11–17].

Learning environments are complex, involving personal, social, organizational, physical, and virtual components [18]. Multiple individuals from different stakeholder groups contribute to the creation of workplace environments, and the attitudes of these individuals may influence an organizations’ culture regarding how and what residents learn [19–23]. Attitudes are also important (albeit imperfect) predictors of individual behavior [24], as evidenced by multiple studies showing associations between physician attitudes and beliefs and their utilization of healthcare services [25–28]. Understanding the attitudes of key stakeholders thus has the potential to offer valuable insights into the post-graduate training environment [29], but there is a scarcity of reliable tools to measure individual attitudes on all dimensions of HVCCC.

In post-graduate medical training, staff physicians, administrators and patients shape residents’ recognition and understanding of HVCCC’s necessity [15, 17, 30–32]. While different stakeholders can have different preferences regarding the provision of HVCCC, measuring all stakeholders’ attitudes can give insight in the resident’s workplace environment regarding the different dimensions of providing HVCCC. Prior studies have tried to measure the attitudes of particular stakeholder groups with respect to specific dimensions of HVCCC [8, 10, 23, 32–39]. However, a single reliable instrument to measure the individual attitudes of all these stakeholder groups toward multiple dimensions of providing HVCCC has not yet been developed. Such an instrument could both assess attitudes at the individual level and compare attitudes between stakeholders on distinct dimensions. It also enables comparisons among different units, organizations, and specialties on the dimensions of providing HVCCC.

This study aims to a) develop an instrument, the Maastricht HVCCC-Attitudes Questionnaire (MHAQ), to measure resident, staff physician, administrator and patient attitudes toward HVCCC and b) determine, using generalizability (G) theory [40], how many respondents are needed to reliably measure a specialty attitude score on a national level.

## Method

We reviewed the literature to identify existing instruments for assessing individual attitudes toward HVCCC. From these, we selected items from the questionnaire used by Leep Hunderfund et al. [36] in their study of medical student attitudes toward cost-conscious care. These items were based on previously published surveys of practicing physicians and focus groups interviews with physicians, who gave input and suggestions on the items, as well as on reviews of the literature on cost-conscious care with input from various field experts [8, 33–35], supporting its content validity [41]. For more details on the development of the items, see the study by Leep Hunderfund et al. [36]. However, the concept of HVCCC consists of three key dimensions. Next to cost-conscious care and potential drawbacks, containing both the direct cost-effectiveness and downstream consequences of including cost-effectiveness, also the provision of value needs to be addressed [7]. Furthermore, because results were reported on an item level, underlying constructs needed to be explored in order to methodologically interpret and compare results of different stakeholders.

**Fig. 1:**
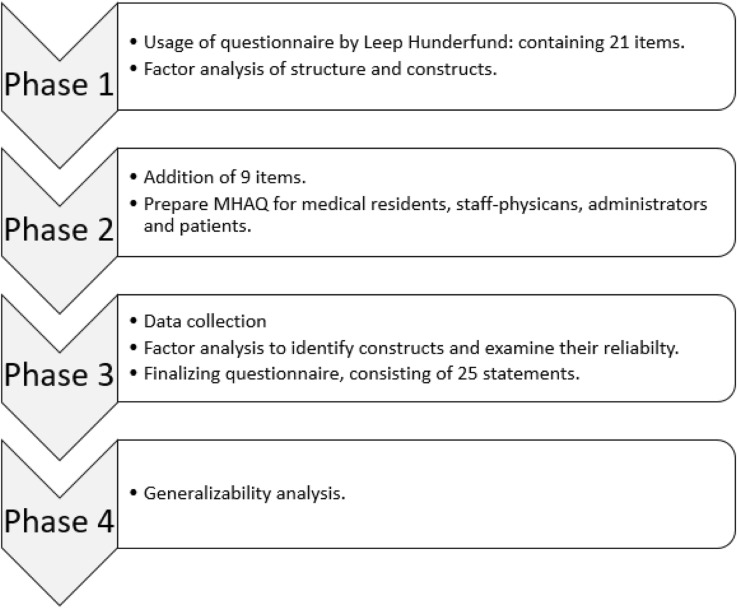
Overview of the four-phase process to develop the MHAQ

We developed the MHAQ through a four-phase process (Fig. 1):

1. Investigating subscales of cost-conscious care, using items and original data from the survey conducted by Leep Hunderfund, et al. [36].
2. Adding items, which include the value dimension, to strengthen subscales, and adapting items for use by residents, staff physicians, administrators, and patients.
3. Testing items among four samples of these stakeholders and developing the final version of the MHAQ.
4. Assessing the number of respondents per specialty on a national level needed to reliably measure a specialty attitude score through generalizability analysis.

### Phase 1: investigating subscales

#### Questionnaire and data

We used items from the aforementioned published survey of U.S. medical students as the starting point for questionnaire development, as this survey derived their 21 items assessing individual attitudes toward cost-conscious care, on recently published surveys for practicing physicians [36]. The authors used a four-point Likert scale (1 = strongly disagree to 4 = strongly agree).

#### Analysis

Since we developed a new scale without having a priori hypotheses about the structure of the variables, we used exploratory factor analysis (principle component analysis, PCA) to examine the structure of these 21 survey items and to define subscales. PCA maximizes explained variance of the items [42] and is considered suitable when examining new constructs [43, 44]. Varimax rotation was performed to maximize spread of all factors, resulting in better interpretable factors [42]. We used a parallel analysis, the Kaiser Guttman criterion (eigenvalues > 1) and inspection of the scree plot, to identify the optimal number of factors [45]. We tested internal-consistency reliability of constructs using Cronbach’s alpha [46].

### Phase 2: preparing the MHAQ

#### Additional items

Based on the internal-consistency reliability of identified subscales (which were around 0.6) and to tailor the MHAQ to new stakeholders and a new context, we added nine items to the original questionnaire. Because the initial 21 items focused primarily on costs, new items focused on value (e.g., risks and benefits of treatment, consideration of patient values) given the importance of value in HVCCC. These items were based on items described in the context of validated surveys on high-value originating from experts in the field [10, 23, 39, 47].

#### Different stakeholders

We developed a parallel questionnaire for medical residents, staff physicians and administrators. Items for patients were identical in content, but formulated for a lay audience. Additionally, we added a fifth answering option (‘I don’t know’) for patients, to prevent random answering when questions were not well understood. These items were pilot-tested with 56 patients in 4 cycles to refine formulations.

#### Different context

For usage in a Dutch context, we translated all items into Dutch. A professional translator translated all items back into English to evaluate similarity between the original source and translated items [48].

### Phase 3: administering the MHAQ and developing the final version

#### Data collection

To recruit respondents, we approached hospital educational committees from all academic training regions (*n* = 8) in the Netherlands. Willing members of the hospital educational committees recruited medical residents and staff physicians to participate in the study. Additionally, we approached residents and staff physicians through the periodic newsletter of the ‘Bewustzijnsproject’, a Dutch project promoting HVCCC on a national level. The last authors (F.S. and L.S.) approached administrators (policy and/or financial) in several hospitals. We approached patients before and after patient consults, after gaining (ethical) approval by the relevant hospital and the physician in charge of the department, and via several patient platforms. We sent all invitations to complete the MHAQ between June 2017 and July 2018. Participants received an information letter, after which they signed an informed consent form before answering the questionnaire. Medical residents, staff physicians and administrators filled out the questionnaire online via Qualtrics, a survey software program. Patients also had the option to answer the questionnaire on hardcopy.

#### Analysis

We analyzed data following the same procedure as in Phase 1. We analyzed data from all stakeholder groups separately, after which an optimal solution was determined through a parallel analysis, as well as examination of each of the scree-plots and the Kaiser-Guttman criterion, followed by an inspection of the factor loadings. We calculated internal consistency reliability of constructs separately for all subscales and all stakeholders using Cronbach’s alpha. Since we developed new scales, a Cronbach’s alpha > 0.6 was considered acceptable [49].

### Phase 4: generalizability analysis

We conducted a generalizability analysis [50] to assess the number of respondents needed to reliably measure a shared attitude score toward HVCCC of residents and staff physicians by specialty on a national level. We used Levene’s homogeneity tests to determine equal variances between specialties of different hospitals. In terms of generalizability theory, we performed a single facet analysis with attitude scores nested within specialties. We carried out a variance component analysis, using specialty as random factor and attitude score as dependent factor. We estimated the variance associated with specialties and the variance of attitude scores nested within specialties using the following formula:

$$ G=\frac{Vs}{Vs+\frac{Vp:s}{Np}} $$

in which Vs is the associated variance of specialties, *Vp:s* is the associated variance of a participants’ attitude score within specialties, and *Np* is the number of participants attitude scores. We used results from G-study variance components to estimate SEM and conduct D-studies to project reliability estimates for varying numbers of respondents. For feasibility, we accepted a G-coefficient greater than 0.6 [50]. All data were analyzed using IBM SPSS statistics for Windows, version 25.0 (Armonk, NY: IBM Corp.).

## Results

### Phase 1

The dataset from the published study on cost-conscious care included responses from students at 10 medical schools geographically distributed across the U.S.. Nine of these schools granted permission to use de-identified data from their students for the purposes of this study (3195 responses of 5992 total students surveyed). No student identifiers were collected and we removed school identifiers prior to sharing. Results of PCA indicated a three subscale-model. All factors had eigenvalues above 1.5. The first subscale contained five items about the responsibility of physicians to provide/promote HVCCC (Table 1); the second subscale contained five items about the relationship of physicians and patients when implementing HVCCC; the final subscale contained four items about considering costs in clinical decision making. Cronbach’s alphas of the subscales were between 0.64 and 0.66. Seven items had factor loadings < .4, representing a low communality for these items, and were not included in these subscales. These items, however, were still included in phases 2 and 3.

**Table 1:** Original items per subscale

Survey item	Cronbach’s alpha
***Subscale 1***	α = .65
Physician clinical practices (e.g., ordering, prescribing) are key drivers of high health care costs. Cost to society should be important in physician decisions to use or not to use an intervention. Cost-effectiveness data should be used to determine what treatments are offered to patients. Trying to contain costs is the responsibility of every physician. Managing health care resources for all patients is compatible with physicians’ obligation to serve individual patients.	
***Subscale 2***	α = .64
Patients will be less satisfied with the care they receive from physicians who discuss costs when choosing tests and treatments. Doctors are too busy to worry about the costs of tests and procedures. It is easier to order a test than to explain to the patient why a particular test is unnecessary. Practicing cost-conscious care will undermine patients’ trust in physicians. Ordering fewer tests and procedures will increase physicians’ risk of medical malpractice litigation.	
***Subscale 3***	α = .66
Physicians should take a more prominent role in limiting use of unnecessary tests. Physicians should be aware of the costs of the tests or treatments they recommend. Physicians should talk to patients about the costs of care when discussing treatment options. Physicians should change their clinical practices (eg, ordering, prescribing) if the cost of care they provide is higher than colleagues who care for similar patients.	

### Phase 2

Table 3 shows the nine new items we added in phase 2, indicated with an asterisk. After translation into Dutch language, content of the original source items and the translated items was identical. The resulting questionnaires for all stakeholder groups contained 30 items, including 21 items from the original questionnaire and nine newly added items.

### Phase 3

In total, 301 residents and 297 staff-physicians completed the MHAQ. Residents and staff physicians worked in 31 different specialties and 32 hospitals, geographically distributed across the Netherlands. Fifty-three administrators and 521 patients completed the MHAQ. Administrators and patients came from five hospitals in the South of the Netherlands (Table 2).

**Table 2:** Demographics of each stakeholder group

Characteristics	Residents	Staff physicians	Administrators	Patients
N respondents	301	297	53	521
N female respondents (%)	191 (65)	151 (51)	27 (51)	241 (46)
Age in years, Mean	30.6	45.9	51.7	59
Medical specialty (%)	296 (98.3)	295 (99.3)	-	-
*Non-Surgical*	172 (57.1)	166 (55.9)	-	-
*Surgical*	89 (29.6)	70 (23.6)	-	-
*Supportive*	35 (11.6)	59 (19.9)	-	-
Type of administrator (%)				
*Department administrator*	-	-	17 (32.1)	-
*Division administrator*	-	-	13 (24.5)	-
*Hospital administrator - Board level*	-	-	7 (13.2)	-
*Other Administrator*	-	-	16 (30.2)	-

#### Data analyses

To develop a questionnaire that is applicable to multiple stakeholders in postgraduate medical education and enables reliable comparisons between stakeholders, grouping of items per subscale has to be the same for all stakeholders. S.M. and K.K. determined a best-fitting subscale composition for all stakeholders, based on the inspection of factor structures for each of the stakeholders. When compromises were necessary, factor analyses of residents and staff-physicians were prioritized when creating optimal subscales for all stakeholders, since these groups are most central in post-graduate medical training. The best-fitting subscale composition for all stakeholders was a three-factor model. All factors had eigenvalues above 1. Four of five items of subscale 1 in phase 1 again clustered on the same factor, together with three additional items from the original subscale 3, as well as two items that had a low factor loading in phase 1 and one new item. The four items of subscale 2 in phase 1 again loaded all on the same factor. Three new items also loaded on this factor. The remaining item from subscale 3 loaded on a third factor, which also included one item from subscale 1, two items with low factor loadings in phase 1, and four new items. Thus, eight of the nine items added in phase 2 strengthened the subscales. All items in phase 1 focused on cost-conscious care, but in phase 3 some of these items loaded on high value care. This is due to the content of these items, which do contain a cost component, but are in essence statements on high value care. Because in phase 1 high value care was not evaluated, these items loaded in this phase on a different subscale. For the final subscale composition, we optimized Cronbach’s alphas for each stakeholder group, considering all subscales had to fit every stakeholder.

#### Final MHAQ

The aforementioned analyses resulted in 25 items distributed among three subscales, each covering an important dimension of HVCCC in clinical environments. We defined the labels of subscales in our team of experts, based on the main focus of the consisting items. Subscale 1, defined as *high-value care*, contained eight items about physicians’ provision of high value care (Cronbach’s alphas ranging from 0.61 for staff physicians to 0.77 for administrators). Subscale 2, defined as *cost incorporation,* contained 10 items about the integration of healthcare costs in physicians’ daily practice (Cronbach’s alphas ranging from 0.69 for staff physicians to 0.80 for patients). Subscale 3, defined as *perceived drawbacks,* contained seven items about perceived drawbacks of practicing HVCCC (Cronbach’s alphas ranging from 0.67 for residents to 0.82 for patients)*.* Table 3 presents the final version of the MHAQ. (The survey instrument is available as supplementary file.)

**Table 3:** An overview of the MHAQ, viewing all items per subscale. (R) Reversed items.

Survey item	Cronbach’s alpha			
	Residents	Staff-physicians	Administrators	Patients
(1) ***High-value care***	α = .65	α = .61	α = .77	α = .67
Physicians should take a more prominent role in limiting use of unnecessary tests. The cost of a test or medication is only important if the patient has to pay for it out of pocket. (R) Managing health care resources for *all* patients is compatible with physicians’ obligation to serve *individual* patients. Eliminating unnecessary tests and procedures will improve patient safety. Physicians should consider a patient’s doubts and values in their clinical decisions.^a^ Physicians should offer patients choices of care, taking advantages, disadvantages and costs into account.^a^ Physicians should limit waste of care in their own hospital/clinic.^a^ Physicians should have sufficient knowledge of the interplay between advantages/disadvantages and costs of common tests.^a^				
(2) ***Cost incorporation***	α = .71	α = .69	α = .74	α = .80
Physicians should try not to think about the cost to the health care system when making treatment decisions. (R) Physicians should be aware of the costs of the tests or treatments they recommend. Physicians should talk to patients about the costs of care when discussing treatment options. Physicians should change their clinical practices (e.g., ordering, prescribing) if the costs of care they provide is higher than colleagues who care for similar patients. Physician clinical practices (e.g., ordering, prescribing) are key drivers of high health care costs. Costs to society should be important in physician decisions to use or not to use an intervention. It is unfair to ask physicians to be cost-conscious and still keep the welfare of their patients foremost in their minds. (R) Cost-effectiveness data should be used to determine what treatments are offered to patients. Trying to contain costs is the responsibility of every physician. Physicians should discuss cost efficiency of care with their patients.^a^				
(3) ***Perceived drawbacks***	α = .67	α = .70	α = .79	α = .82
Patients will be less satisfied with the care they receive from physicians who discuss costs when choosing tests and treatments. Doctors are too busy to worry about the costs of tests and procedures. Practicing cost-conscious care will undermine patients’ trust in physicians. Ordering fewer tests and procedures will increase physicians’ risk of medical malpractice litigation. Ordering more tests reduces a physicians’ diagnostic uncertainty.^a^ Ordering fewer tests and procedures will lead to more complications.^a^ Patients find it unpleasant to talk about costs of tests or treatments.^a^				

^a^New items that were added in phase 2. The item “if a physicians’ medical practices have a direct influence on a physicians’ salary, it will obstruct a physicians’ cost-conscious care approach” did not cluster on any of the subscales

### Phase 4

#### Generalizability

**Fig. 2:**
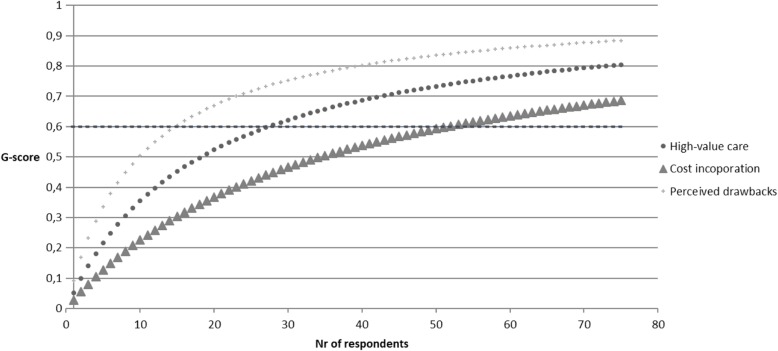
D-study projecting MHAQ reliability of resident respondents*. Note: value of 0.6 is considered reliable*

**Fig. 3:**
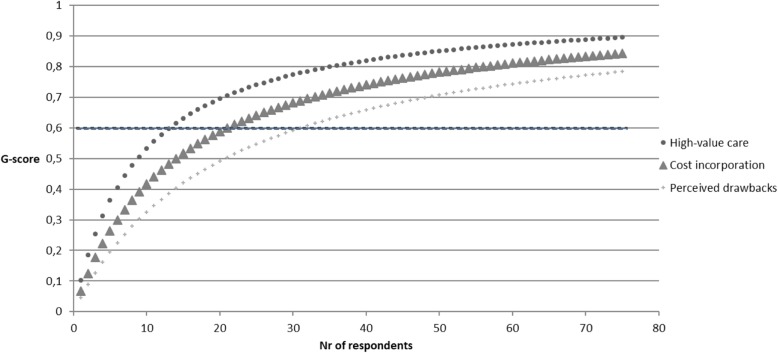
D-study projecting MHAQ reliability of staff physician respondents*. Note: value of 0.6 is considered reliable*

This reliability estimation was performed separately for medical residents and staff physicians and for each subscale. Levene’s homogeneity tests indicated equal variances between specialties (e.g., cardiology, internal medicine) across different hospitals. Results from D-studies indicated the number of respondents needed to reliably measure (G-score ≥ 0.6) residents’ attitude score per specialty on a national level is 28 for the subscale high value care, 52 for the subscale cost incorporation, and 15 for the subscale perceived drawbacks. For staff physicians, the number of respondents needed was respectively 14 for the subscale high value care, 21 for the subscale cost incorporation, and 32 for the subscale perceived drawbacks. Figures 2 and 3 display an overview of the G-score per subscale for residents and staff physicians.

## Discussion

This study describes the development of the MHAQ and provides reliability evidence supporting its use to measure attitudes toward HVCCC among important stakeholders in the post-graduate clinical learning environment. The MHAQ assesses three key dimensions of HVCCC and may be used to identify frontrunners who endorse and prioritize HVCCC, to pinpoint aspects of HVCCC that need to be improved or changed to better support HVCCC in the post-graduate learning environment, and to facilitate comparisons among different stakeholder groups, specialties, regions, and potentially hospitals or departments. The MHAQ includes three subscales relating to provision of high-value care (8 items), integration of costs (10 items), and perceived drawbacks of HVCCC (7 items). These subscales encompass all key dimensions of providing HVCCC in clinical practice [7], hence supporting the content validity of MHAQ scores.

Scores on high-value care reflect the degree to which individuals believe physicians should be responsible for limiting unnecessary testing, reducing waste, considering risks, benefits, and patient preferences when making diagnostic or therapeutic intervention decisions. High scores on this subscale can identify proponents of HVCCC who believe physicians should be frontrunners in the provision of high-value care. When key individuals within the clinical learning environment advocate high-value care, corresponding role modelling can help to shape future physicians’ HVCCC practice patterns [17, 30, 51].

Scores on cost incorporation reflect individual beliefs about the degree to which physicians should integrate costs in their daily clinical practice, for example when making treatment decisions or when discussing options with patients. Although physicians assume they contribute minimally to healthcare costs [35], they actually direct up to 87% of all healthcare spending [52]. Knowing physicians’ view on the incorporation of costs in their daily practice, together with patients’ view on the incorporation of costs, can be important starting points for transformation efforts to educate future physicians about providing HVCCC [14].

Scores on perceived drawbacks reflect individual beliefs about potential drawbacks of HVCCC, like patient dissatisfaction or risks of malpractice. Perceptions like these are known barriers to the implementation of HVCCC in practice [53] and drivers of unnecessary testing [54]. When individuals within the same organization have different perceptions of the drawbacks, incorporation of HVCCC in daily clinical practices is unsustainable. Pinpointing organizations as such could initiate aligned education programs for all stakeholders in that organization on the benefits of HVCCC, to create a common understanding and support of the delivery of HVCCC [17, 55].

Internal consistency reliability was sufficient for all stakeholders on all subscales. The internal consistency reliability for subscale scores was lower for residents and staff physicians than for patients and administrators. This could suggest that residents and physicians have more nuanced views on the provision of high-value care, integration of costs into clinical practice, and potential drawbacks of HVCCC. Alternatively, items formulated for a lay audience may be more evident in meaning and therefore clearer to answer than items used in the questionnaires for residents, staff physicians, and administrators. The patient version of the MHAQ thus has the potential to inform future improvement of subscale reliability for other stakeholders when developing the MHAQ further.

The MHAQ can not only be used to measure attitudes toward HVCCC at the individual level, but also to compare attitudes among larger groups, e.g. specialties, hospitals, regions. Our D-study results predict 14 to 52 respondents would be required to reliably assess HVCCC attitudes among resident or staff physicians, supporting the feasibility of group comparisons at the national, specialty level.

### Strengths and limitations

This study has certain strengths and limitations. First, the MHAQ is based on a previously published questionnaire informed by a literature review on HVCCC, which was further enhanced through the addition of items (also based on the literature) that emphasized value as an important dimension in addition to cost and drawbacks. Future studies could provide additional content validity evidence for MHAQ scores by presenting items to subject matter experts, for example in a Delphi-study [56]. Second, while we are the first, to our knowledge, to simultaneously survey resident, staff physician, administrator, and patient attitudes toward HVCCC, our study did not include all potential stakeholders. Future studies could extend our work by including other relevant groups, such as nurses and other allied health professionals, who contribute to the clinical learning environment. Third, we used the same items in the U.S. and the Netherlands, which strengthens the broad usability of the MHAQ. However, healthcare delivery systems vary by country and MHAQ items may not be equally applicable in all settings. Fourth, while the final version of the MHAQ showed promising reliabilities, and D-studies support the feasibility of reliable assessments at the specialty level, there were too few results from a single department within a single hospital to calculate a reliable G-score at the department level. Further studies are needed to assess the number of respondents needed for a reliable department-level attitude score, which may most closely approximate the clinical learning environment experience by residents.

## Conclusion

The MHAQ is a new instrument capable of reliably measuring attitudes toward HVCCC among individuals within multiple relevant stakeholder groups - residents, staff physicians, administrators, and patients - with subscales that address key dimensions of HVCCC. The MHAQ can be used to identify frontrunners who endorse and prioritize HVCCC, to pinpoint aspects of HVCCC that need to improved or changed to better support HVCCC in the post-graduate learning environment, and to facilitate comparisons among different stakeholder groups, specialties, regions, and potentially hospitals or departments.

## Supplementary information

**Additional file 1.** The Maastricht HVCCC Attitude Questionnaire (MHAQ).

## Data Availability

The Dutch dataset collected during the current study is available from the corresponding author on reasonable request.

## Abbreviations

D-studies: Decision studies
G-coefficient: Generalizability coefficient
G-score: Generalizability score
G-studies: Generalizability studies
HVCCC: High-Value, Cost Conscious Care
MHAQ: Maastricht HVCCC Attitude Questionnaire
PCA: Principal Component Analysis
SEM: Standard Error of Measurement
U.S.: United States
