# Supplementary material for: A new instrument to measure high value, cost-conscious care attitudes among healthcare stakeholders: development of the MHAQ
Source: BMC Health Serv Res. 2020 Mar 2;20:156. doi: 10.1186/s12913-020-4979-z (PMC7053044; doi:10.1186/s12913-020-4979-z)
Supplement: Supplementary file 1 — Additional file 1. The Maastricht HVCCC Attitude Questionnaire (MHAQ). [file 12913_2020_4979_MOESM1_ESM.docx]

**The Maastricht HVCCC Attitude Questionnaire (MHAQ)**

Mordang, S. B. R., Könings, K. D., Leep Hunderfund, A. N., Paulus, A. T. G., Smeenk, F. W. J. M., & Stassen, L. P. S. A new instrument to measure high value, cost-conscious care attitudes among healthcare stakeholders: development of the MHAQ.

Indicate how much you agree or disagree with each of the following statements:

|  | Strongly disagree | Disagree | Agree | Strongly agree |
| --- | --- | --- | --- | --- |
| 1. Clinicians should take a more prominent role in limiting use of unnecessary tests. | ○ | ○ | ○ | ○ |
| 2. Physicians should try not to think about the cost to the health care system when making treatment decisions. | ○ | ○ | ○ | ○ |
| 3. Physicians should be aware of the costs of the tests or treatments they recommend. | ○ | ○ | ○ | ○ |
| 4. Physicians should talk to patients about the costs of care when discussing treatment options. | ○ | ○ | ○ | ○ |
| 5. Physicians should change their clinical practices (e.g., ordering, prescribing) if the costs of care they provide is higher than colleagues who care for similar patients. | ○ | ○ | ○ | ○ |

Indicate how much you agree or disagree with each of the following statements:

|  | Strongly disagree | Disagree | Agree | Strongly agree |
| --- | --- | --- | --- | --- |
| 6. Physician clinical practices (e.g., ordering, prescribing) are key drivers of high health care costs. | ○ | ○ | ○ | ○ |
| 7. Cost to society should be important in physician decisions to use or not to use an intervention. | ○ | ○ | ○ | ○ |
| 8. The cost of a test or medication is only important of the patient has to pay for it out of pocket. | ○ | ○ | ○ | ○ |
| 9. It is unfair to ask physicians to be cost-conscious and still keep the welfare of their patients foremost in their minds. | ○ | ○ | ○ | ○ |
| 10. Cost-effectiveness data should be used to determine what treatments are offered to patients. | ○ | ○ | ○ | ○ |
| 11. Patients will be less satisfied with the care they receive from physicians who discuss costs when choosing tests and treatments. | ○ | ○ | ○ | ○ |
| 12. Doctors are too busy to worry about the costs of tests and procedures. | ○ | ○ | ○ | ○ |

Indicate how much you agree or disagree with each of the following statements:

|  | Strongly disagree | Disagree | Agree | Strongly agree |
| --- | --- | --- | --- | --- |
| 13. Trying to contain costs is the responsibility of every physician. | ○ | ○ | ○ | ○ |
| 14. Managing health care resources for *all* patients is compatible with physicians’ obligation to serve *individual* patients. | ○ | ○ | ○ | ○ |
| 15. Eliminating unnecessary tests and procedures will improve patient safety. | ○ | ○ | ○ | ○ |
| 16. Practicing cost-conscious care will undermine patients’ trust in physicians. | ○ | ○ | ○ | ○ |
| 17. Ordering fewer tests and procedures will increase physicians’ risk of medical malpractice litigation. | ○ | ○ | ○ | ○ |

Indicate how much you agree or disagree with each of the following statements:

|  | Strongly disagree | Disagree | Agree | Strongly agree |
| --- | --- | --- | --- | --- |
| 18. Physicians should consider a patient’s doubts and values in their clinical decisions. | ○ | ○ | ○ | ○ |
| 19. Physicians should offer patients choices of care, taking advantages, disadvantages and costs into account. | ○ | ○ | ○ | ○ |
| 20. Physicians should limit waste of care in their own hospital/clinic. | ○ | ○ | ○ | ○ |
| 21. Ordering more tests reduces a physicians’ diagnostic uncertainty. | ○ | ○ | ○ | ○ |
| 22. Physicians should have sufficient knowledge of the interplay between advantages/disadvantages and costs of common tests. | ○ | ○ | ○ | ○ |
| 23. Ordering fewer tests and procedures will lead to more complications. | ○ | ○ | ○ | ○ |
| 24. Patients find it unpleasant to talk about costs of tests or treatments. | ○ | ○ | ○ | ○ |
| 25. Physicians should discuss cost efficiency of care with their patients. | ○ | ○ | ○ | ○ |
